# Quantitative analysis of tumor-specific BCL2 expression in DLBCL: refinement of prognostic relevance of BCL2

**DOI:** 10.1038/s41598-020-67738-4

**Published:** 2020-06-30

**Authors:** Jin Roh, Hyungwoo Cho, Dok Hyun Yoon, Jung Yong Hong, A-Neum Lee, Hyeon Seok Eom, Hyewon Lee, Weon Seo Park, Jae Ho Han, Seong Hyun Jeong, Joon Seong Park, Hyo-Kyung Pak, So-Woon Kim, Sang-Yeob Kim, Cheolwon Suh, Jooryung Huh, Chan-Sik Park

**Affiliations:** 1 Department of Pathology, Ajou University School of Medicine, Suwon, Republic of Korea; 2 Department of Oncology, Asan Medical Center, University of Ulsan College of Medicine, Seoul, Republic of Korea; 3 Asan Institute for Life Science, Asan Medical Center, University of Ulsan College of Medicine, Seoul, Republic of Korea; 4 Center for Hematologic Malignancy, National Cancer Center, Goyang, Republic of Korea; 5 Department of Hematology-Oncology, Ajou University School of Medicine, Suwon, Republic of Korea; 6 Department of Pathology, Asan Medical Center, University of Ulsan College of Medicine, Seoul, Republic of Korea; 7 Department of Convergence Medicine, University of Ulsan College of Medicine, Seoul, Republic of Korea

**Keywords:** Cancer, Lymphoma

## Abstract

BCL2 overexpression has been reported to be associated with poor prognosis in patients with diffuse large B-cell lymphoma (DLBCL). However, currently there is no consensus on the evaluation of BCL2 expression and only the proportion of BCL2 positive cells are evaluated for the determination of BCL2 positivity. This study aimed to define BCL2 positivity by quantitative analysis integrating both the intensity and proportion of BCL2 expression. BCL2 expression of 332 patients (221 patients for the training set and 111 patients for the validation set) with newly diagnosed DLBCL who received R-CHOP (rituximab, cyclophosphamide, doxorubicin, vincristine, and prednisone) were analyzed using the tumor-specific automated quantitative analysis (AQUA) scoring method based on multiplex immunofluorescence. In the training set, high BCL2 AQUA score (*N* = 86, 38.9%) was significantly associated with poor prognosis (*p* = 0.01, HR 2.00; 95% CI [1.15–3.49]) independent of international prognostic index, cell of origin, and MYC expression. The poor prognostic impact of the high BCL2 AQUA score was validated in the validation set. AQUA scoring of BCL2 expression incorporating both the intensity and proportion of BCL2 positive cells was independently associated with survival outcomes of patients with primary DLBCL treated with R-CHOP.

## Introduction

Diffuse large B-cell lymphoma (DLBCL) is the most common type of non-Hodgkin lymphoma which is characterized by a variety of molecular aberrations^1^. The survival outcome of DLBCL patients has improved with the addition of anti-CD20 monoclonal antibody rituximab to cyclophosphamide, doxorubicin, vincristine, and prednisone (R-CHOP)^2^. However, approximately 40% of patients are refractory to R-CHOP and/or eventually experience disease progression^3^. Although the International Prognostic Index (IPI) remains as a valid prognostic index for patients with DLBCL in the R-CHOP era^4^, it has suboptimal predictive value in discriminating high-risk patients and does not reflect the underlying tumor biology. The cell of origin (COO) was introduced to reflect the biological characteristics of DLBCL; however, its prognostic value still remains controversial^5^. In addition to the COO-based approach, more recent studies have reported that DLBCL with BCL2 and MYC abnormalities, such as double-expressor and double-hit lymphoma, is associated with more aggressive clinical course and a poor survival outcome^6–9^.

BCL2 is one of the most important anti-apoptotic protein and is essential for B-cell development and maturation^10–13^. Various mechanisms of *BCL2* deregulation, including amplification, translocation, and overexpression are associated with the development of cancer^14^. *BCL2* was initially discovered as a genetic hallmark of follicular lymphoma^10^ and contributes to the pathogenesis of various hematologic malignancies including DLBCL^15^. In previous DLBCL studies, highly variable (24–80%) BCL2 positivity rates were observed, which would be mainly due to subjective and semiquantitative interpretation and the absence of the established cutoff value for BCL2 expression by immunohistochemistry (IHC). Consequently, the clinical implication of BCL2 varies between studies^7,8,16–24^. However, a recent study reported that a semiquantitative BCL2 IHC scoring system incorporating both proportion and intensity had strong independent prognostic power in patients with DLBCL treated with R-CHOP^25^.

Here, we investigated tumor-specific BCL2 expression with the automated quantitative analysis (AQUA) scoring system using the multiplex immunofluorescent (IF) imaging to assess the prognostic impact of quantitative BCL2 expression in patients with newly diagnosed DLBCL treated with R-CHOP.

## Materials and methods

### Patients, samples, and tissue microarray (TMA)

We retrospectively collected formalin-fixed, paraffin-embedded (FFPE) diagnostic biopsies from 221 patients with primary DLBCL between 2007 and 2012 at Asan Medical Center. All patients underwent standard staging procedures and treatment with R-CHOP. Patients with primary central nervous system (CNS) lymphoma or with treatments other than R-CHOP were excluded. Clinical information was obtained from medical records including sex, age, treatment regimens, serum LDH level, presence of B symptoms, treatment response, serum hemoglobin level, Ann Arbor stage, IPI, and COO. COO was determined using IHC according to the Hans classification^26^. Among the patients, information regarding COO was available in 207 cases. An independent validation set included 111 patients diagnosed as primary DLBCL between 2010 and 2012 at Ajou University Hospital and between 2005 and 2016 at National Cancer Center in Korea. Patient selection and TMA construction were performed in the same manner. The protocols of this study were approved by the Institutional Review Board (IRB) of Asan Medical Center (2019IP0408) and Ajou University Medical Center (AJIRB-MED-SMP-18-259), and was allowed to waive the requirement to obtain informed consent. All experiments and methods were performed in accordance with relevant guidelines and regulations.

All H&E-stained slides were reviewed to ensure an accurate diagnosis and that the appropriate amount of tumor remained. When multiple blocks were available for any single case, one or two blocks containing representative tissue for the TMA were selected. Cases with an insufficient amount of tumor for TMA were excluded from the study cohort. The TMAs contained at least two representative 1–1.5 mm cores from each tumor to represent conventional sections^27^.

### Multiplex IF and quantitative AQUA scoring

The methods described below have been reproduced in part from our recent publication^28^. Briefly, 4 µm-thick sections were deparaffinized in xylene and dehydrated in graded ethanols. Antigen retrieval was performed in citrate buffer (pH 6.0) with microwave heating. Primary antibodies were as follows: CD20 (clone L26; Dako, CA, USA), CD3 (polyclonal [cat. A0452]; Dako, CA, USA), BCL2 (clone 124; Dako, CA, USA), and MYC (clone Y69; Abcam, CB, UK). Envision + poly-HRP-anti-mouse and Envision + poly-HRP-anti-rabbit (Dako, CA, USA) were used as secondary antibodies. Staining was optimized by performing a duplex (CD20—Opal 650 and BCL2—Opal 520), followed by a triplex (addition of CD3—Opal 570). All multiplex experiments were performed by repeating staining cycles in series, with microwave treatments between each cycle and at the end of the experiment. All multiplexed stains were finished with 4′,6-Diamidino-2-Phenylindole (DAPI) counterstaining. All stained slides were scanned using the Vectra automated quantitative pathology imaging system (Vectra 3.0.3; PerkinElmer, MA, USA) and analyzed using the InForm Advanced Image Analysis software (InForm 2.2.1; PerkinElmer, MA, USA). Multispectral images obtained by scanning were unmixed using spectral libraries built from images of single stained tissues for each reagent. Each cell was identified by detecting nuclear spectral element (DAPI) and the specific fluorescent spectra within every subcellular compartment was analyzed (CD20—membrane, CD3—cytoplasm, BCL2—cytoplasm, MYC—nucleus) (Supplementary Figure S1). The fluorescent intensities for each marker were quantified on a per-pixel basis and were normalized between 0 and 1.

To analyze tumor-specific biomarker expression, tumor cells were selected using CD20 expression. All images were confirmed by pathologists (J.R. and C.-S.P.) for optimal staining by manual inspection. The tumor-specific quantified intensity information in pixels was converted to protein expression information on a cell basis using the AQUA scoring system. The AQUA scores for BCL2 and MYC were calculated as the summation of the intensity of corresponding biomarker of each pixel in the target compartment divided by summation of each pixel area of the target compartment (∑intensity of each pixel in the target compartment/∑pixel area of the target compartment)^29^. To additionally evaluate the individual prognostic value of BCL2 intensity and proportion of BCL2 positive cell, we defined BCL2 positive cell as tumor cell with mean cytoplasmic BCL2 intensity ≥ 25th percentile of mean cytoplasmic BCL2 intensity of tumor cells in the target compartment of patients in the training set. Proportion of BCL2 positive cells within a patient was defined as number of BCL2 positive cells divided by total tumor cell count in the target compartment.

### Single chromogenic IHC and quantified image analysis

Single chromogenic IHC for BCL2 was performed using the Bench Mark XT (Ventana Medical Systems, AZ, USA) according to the manufacturer’s instructions. Primary antibodies were the same as those used in multiplex IF staining. After reacting with secondary antibodies, antibody binding was visualized after incubation in diaminobenzidine (DAB) solution for 1 min. All stained slides were scanned with the Pannoramic 250 Flash III (3DHISTECH, BUD, HU) and were analyzed using Pannoramic Viewer software (3DHISTECH, BUD, HU).

The region of interest was manually selected within the TMA scanned image and extracted as 2–6 JPEG images in each case. For quantitative image assessment, single cell segmentation and quantification of staining intensity were performed using a CellProfiler v2.2.0 pipeline, BCL2_analysis.cpproj (https://github.com/jinn0208/BCL2_analysis.git). The thresholds for staining intensity were determined after three pathologists reviewed the entire quantified results (J.R, C-S.P, and S-W.K). For single chromogenic IHC, BCL2 expression was also analyzed using the conventional method in which any BCL2-expressing tumor cells were classified as positive regardless of their intensity. The cutoff value of 50% for BCL2 was used in the conventional analysis. To incorporate the staining intensity to the BCL2 interpretation as in the AQUA scoring system, the H-score was calculated using the following formula: [$1{ } \times \left(\% {{\text{cells }}\;1 + } \right) + 2 \times \left( {\% {\text{cells}} \;2 + } \right) + 3 \times \left( {\% {\text{cells}} \;3 + } \right)$]^30^.

### Statistical analysis

The statistical methods described below have been reproduced in part from our recent publication^28^. Overall survival (OS) was defined as the time from diagnosis until death from any cause. Event-free survival (EFS) was defined as the time from diagnosis until relapse or progression, unplanned re-treatment of lymphoma after initial immunochemotherapy, or death from any cause. Baseline characteristics were compared between the groups using the Student’s *t*-test for continuous variables and the Fisher’s exact test or Pearson’s chi-squared test for categorical variables, as appropriate. The Kaplan–Meier method was used to calculate OS and EFS, which were compared using the log-rank test. Univariate and multivariate Cox proportional hazard regression models were performed to analyze the prognostic value of BCL2 and MYC expression on OS or EFS. Proportional hazard assumptions were confirmed with visual inspection, in which we confirmed that the graph of the log (− log[survival]) versus log of survival time graph resulted in parallel lines^31^.

Calculated AQUA scores and H-score were normalized between 0 and 100 for comparison. ROC curve analysis was implemented to determine the optimized cutoff value and to compare the performances of the different analysis methods. In addition, batch effects within the training and validation set were adjusted by a two-stage regression approach (ber package). All statistical calculations were conducted using R version 3.4.0. (R Foundation for Statistical Computing, https://www.R-project.org/).

## Results

### Quantitative IF in patients with DLBCL showed various BCL2 expression within and between tumors

**Figure 1:**
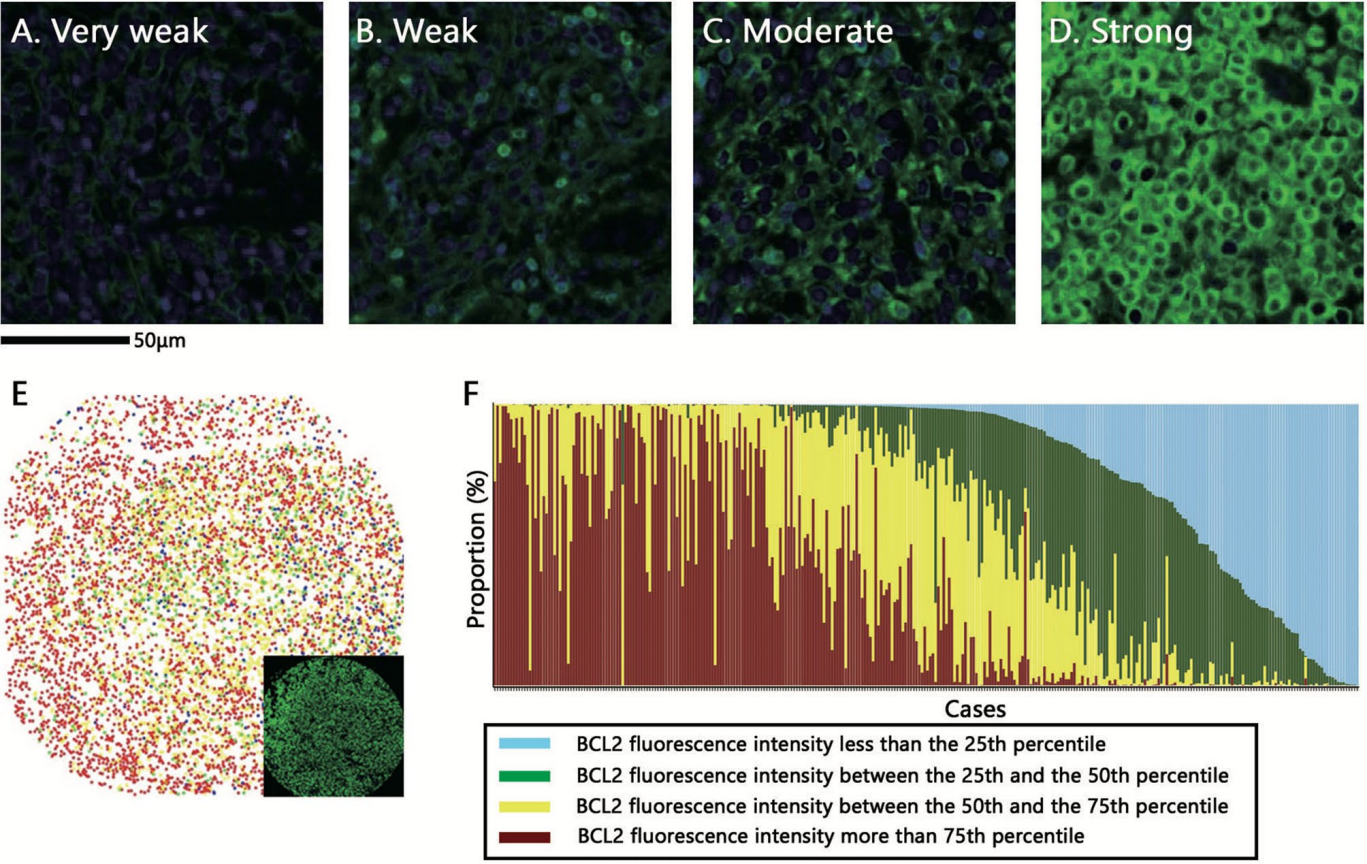
Distribution of BCL2 fluorescence intensities of tumor cells. (**A**, **D**) Representative images of various BCL2 fluorescence intensities regardless of the proportion of immuno-stained tumor cells are shown. The BCL2 fluorescence signal is pseudocolored in green. (**E**, **F**) The BCL2 fluorescence intensities of CD20-positive tumor cells were classified into 4 quantile groups regardless of the case. Subsequently, the mean value of pixel fluorescence intensity per each tumor cell is plotted on a single cell level in a representative core (the inset shows immunofluorescent stained image of the corresponding core) (**E**). The proportion of the cells corresponding to each quantile group is plotted on a case-by-case basis (**F**).

The characteristics of the 221 patients of the training set from Asan Medical Center are shown in Table 1, and those of the 111 patients of the validation set from other institutions in Supplementary Table S1. In the training set, 5-year OS and EFS were 69.0% and 62.2%, respectively with a median follow up of 59 months. The distribution of BCL2 fluorescence intensities of tumor cells is shown in Fig. 1. Regardless of the proportion, various fluorescence intensities of BCL2 were observed among the different cases (Fig. 1A–D). The heterogeneity in intensity within a case was visualized by plotting the BCL2 fluorescence intensity of individual tumor cells on a case-by-case basis (Fig. 1E, F). The mean value of pixel fluorescence intensity per tumor cell ranged 0–317.321 (median 11.504, mean 25.739 ± 30.67). In single chromogenic BCL2 IHC which is commonly used in clinical practice, various chromogenic intensities were also observed (Supplementary Figure S2). Based on these results, we identified that the intensity of BCL2 expression by tumor cells in DLBCL varies widely regardless of its proportion.

**Table 1:** Baseline characteristics of patients with diffuse large B-cell lymphoma in the training set.

Characteristics	Total n = 221 (%)	BCL2 AQUA score			MYC AQUA score		
		Low n = 135 (%)	High n = 86 (%)	*p* value	n = 166 (%)	n = 55 (%)	*p* value
Age > 60 years	98 (44.3)	58 (43.0)	40 (46.5)	0.605	62 (37.3)	36 (65.5)	0.0003
Sex, male	126 (57.0)	77 (57.0)	49 (57.0)	0.9930	97 (58.4)	29 (52.7)	0.4588
LDH > normal	101 (45.7)	59 (43.7)	42 (48.8)	0.455	67 (40.4)	34 (61.8)	0.0056
B symptoms+	58/219 (26.5)	29/133 (21.8)	29 (33.7)	0.0510	42/165 (25.5)	16/54 (29.6)	0.5462
	NA (2)	NA (2)			NA (1)	NA (1)	
Hb < normal	109 (49.3)	61 (45.2)	48 (55.8)	0.1233	79 (47.6)	30 (54.5)	0.3712
Stage 3–4	126 (57.0)	74 (54.8)	52 (60.5)	0.408	93 (56.0)	33 (60.0)	0.6057
IPI 3–5	81 (36.7)	44 (32.6)	37 (43.0)	0.1167	56 (33.7)	25 (45.5)	0.1180
BM involvement	45/220 (20.5)	28 (20.7)	17/85 (20.0)	0.895	34 (20.5)	11/54 (20.4)	0.9859
	NA (1)		NA (1)			NA (1)	
CR	195 (88.2)	126 (93.3)	69 (82.2)	0.003	154 (92.8)	41 (74.5)	0.0003
2-years EFS rate (%)	70.34	80.93	54.04		75.36	55.02	
Non-GCB type	142/207 (68.6)	78/126 (61.9)	64/81 (79.0)	0.01	106/156 (67.9)	36/51 (70.6)	0.7244
	NA (14)	NA (9)	NA (5)		NA (10)	NA (4)	

*LDH* lactate dehydrogenase, *IPI* International Prognostic Index, *CR* complete response, *EFS* event-free survival, *AQUA* automated quantitative analysis, *NA* not available.

### High BCL2 and MYC AQUA scores were associated with poor prognosis

Considering the diversity of BCL2 intensity, we used the AQUA scoring system to generate metrics incorporating an expression intensity in addition to proportion. In the training set, the BCL2 AQUA score was significantly high in various clinical conditions including no achievement of complete response (CR) for initial chemotherapy (*p* = 0.01053), death in the entire observation time (*p* = 0.0008803), death within 5 years of diagnosis (*p* = 0.0008853), and clinical events within 2 years of diagnosis (*p* = 0.000316) (Supplementary Figure S3). According to these results, we can infer that high BCL2 AQUA score is associated with poor clinical conditions. Then, patients were grouped according to the 10th, 50th, and 90th quantile of the BCL2 AQUA score. It showed the worse OS and EFS with increasing the BCL2 AQUA scores (Supplementary Figure S4A,B). This tendency was also observed as increases in hazard rate according to increases in the BCL2 AQUA score in the forest plot (Supplementary Figure S4C,D). In addition, prognostic value of individual BCL2 intensity and proportion of BCL2 positive cells were compared with BCL2 AQUA score as continuous variable by univariate analysis. Although both proportion of BCL2 positive cells and BCL2 AQUA score were significantly associated with OS and EFS, hazard ratio (HR) was higher for BCL2 AQUA score (Supplementary Table S2).

**Figure 2:**
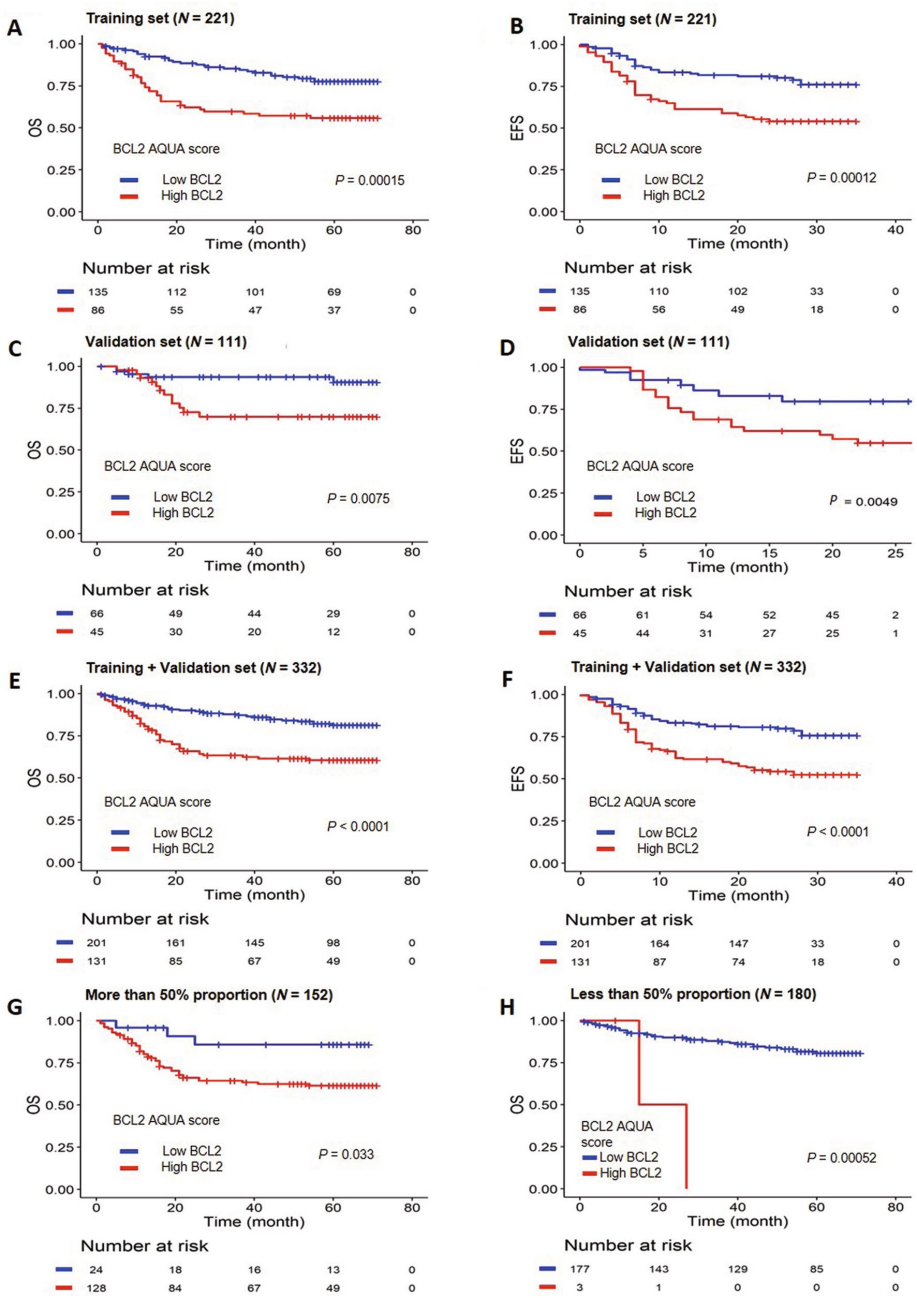
Survival analysis according to the patient groups classified using the optimal cutoff value of the BCL2 Automated Quantitative Analysis (AQUA) score. (**A**, **B**) Overall survival (OS) (**A**) and event-free survival (EFS) (**B**) according to the BCL2 AQUA score in the training set. (**C**, **D**) OS (**C**) and EFS (**D**) according to the BCL2 AQUA score in the validation set. (**E**, **F**) OS (**E**) and EFS (**F**) in the entire cohorts according to the BCL2 AQUA score. (**G**, **H**) OS according to the BCL2 AQUA score within the conventionally classified as the high BCL2 expression group (**G**) and as the low BCL2 expression group (**H**).

The BCL2 AQUA score of 41.47 was determined as the optimal cutoff value in the training set using the ROC analysis. Eighty-six patients (38.9%) in the training set were classified in the high BCL2 group according to the determined cutoff value. Achieving an initial CR and non-germinal center B cell (GCB) type were found to be significantly associated with high BCL2 AQUA score (*p* = 0.0032 and 0.0096, respectively). Other clinicopathologic factors were not associated with the status of the BCL2 AQUA score (Table 1). The high BCL2 group in the training set was significantly associated with poor OS and EFS (*p* = 0.00015; OS, *p* = 0.00012; EFS) (Fig. 2A,B).

To validate the prognostic impact of the cutoff value, the patients in the validation set were classified using the same cutoff BCL2 AQUA score as that of the training set. Forty-five patients among 111 patients in the validation set (40.5%) were classified as being in the high BCL2 group. The high BCL2 group in the validation set was also significantly associated with inferior OS and EFS (*p* = 0.0075; OS, *p* = 0.0049; EFS) (Fig. 2C,D). In addition, we additionally evaluated the prognostic value of BCL2 AQUA score as a continuous variable by univariate analysis and it was significantly associated with both OS (HR: 1.02, *p* = 0.033) and EFS (HR: 1.02, *p* = 0.006).

This poor prognostic impact of BCL2 was also in good correlation with both OS and EFS in the entire cohort (*p* < 0.0001; OS, *p* < 0.0001; EFS) (Fig. 2E,F). Then, we compared the outcomes of these patients using the conventional method and the AQUA score, respectively. Twenty-four of 152 patients classified as being in the high BCL2 group by the conventional method were reclassified as being in the low BCL2 group by the AQUA scoring. These reclassified patients showed a significantly favorable outcome (*p* = 0.033; OS). In addition, 3 of 180 patients conventionally grouped as the low BCL2 group were reclassified as being in the high BCL2 group by AQUA scoring. These reclassified patients showed a relatively inferior outcome (*p* = 0.00052; OS) (Fig. 2G,H).

**Figure 3:**
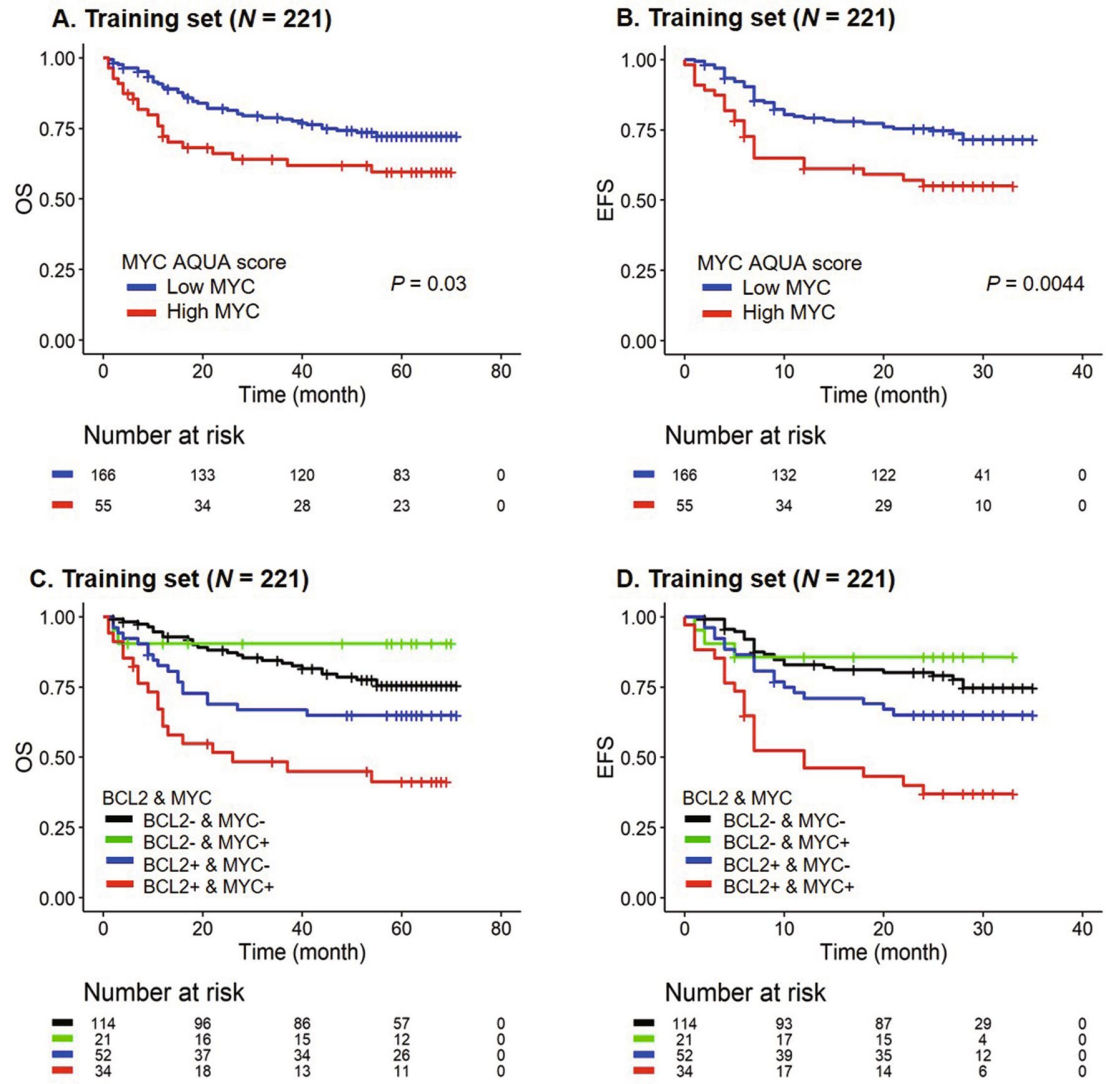
Survival analysis according to the MYC automated quantitative analysis (AQUA) scores and the combined BCL2 and MYC AQUA scores. (**A**, **B**) Overall survival (OS) (**A**) and event-free survival (EFS) (**B**) according to the MYC AQUA score in the training set. (**C**, **D**) OS (**C**) and EFS (**D**) of the patients in the training set based on the combined BCL2 and MYC AQUA scores.

We also calculated the MYC AQUA score in the training set. The MYC AQUA score of 48.83 was determined as the optimal cutoff value. Fifty-five (24.9%) patients were classified as high MYC group. Among clinical features, high LDH level and achieving an initial CR were found to be significantly associated with high MYC AQUA score (*p* = 0.0056 and 0.0003, respectively). High MYC AQUA score was significantly associated with poor OS and EFS (*p* = 0.03; OS, *p* = 0.0044; EFS) (Fig. 3A,B). Next, we classified patients in 4 groups according to the BCL2 and MYC AQUA score groups. Of all these patients, 34 patients (15.4%) showed both high BCL2 and MYC AQUA scores. Patients with both BCL2 and MYC high AQUA scores showed the worst outcome (Fig. 3C,D).

Taken together, we identified that a high BCL2 AQUA score is significantly associated with inferior outcome and patients with both high BCL2 and high MYC AQUA score showed the most inferior outcomes.

### BCL2 AQUA score was an independent poor prognostic factor in DLBCL

**Figure 4:**
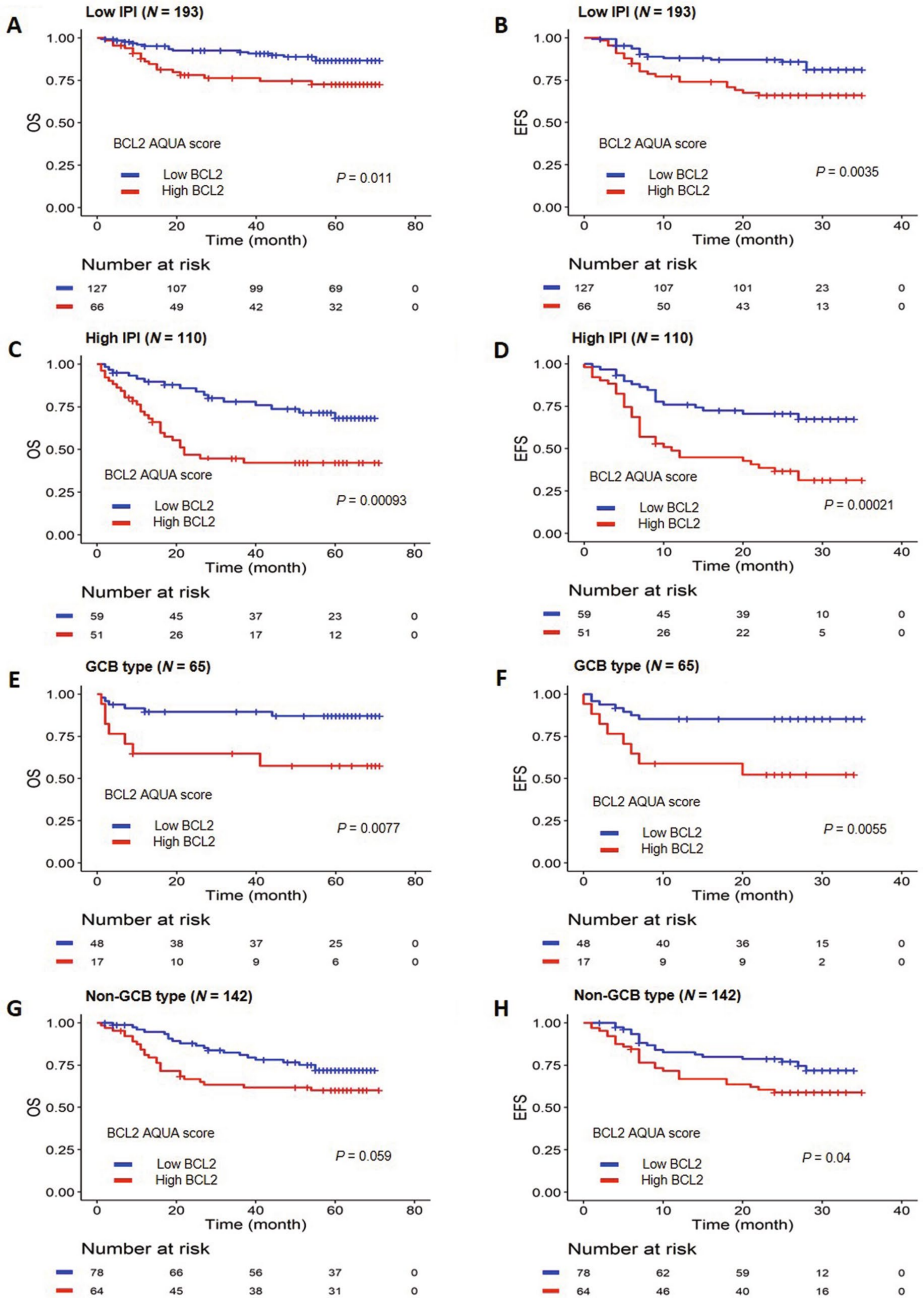
Survival analysis according to the BCL2 automated quantitative analysis (AQUA) score and clinicopathologic factors in the entire cohorts. (**A**, **B**) Overall survival (OS) (**A**) and event-free survival (EFS) (**B**) within the low International Prognostic Index (IPI) group. (**C**, **D**) OS (**C**) and EFS (**D**) within the high IPI group. (**E**, **F**) OS (**E**) and EFS (**F**) within the germinal center B-cell (GCB) type of diffuse large B-cell lymphoma (DLBCL). (**G**, **H**) OS (**G**) and EFS (**H**) within the non-GCB type of DLBCL.

We performed additional survival analysis according to the various clinicopathologic factors. First, we analyzed the relationship between the BCL2 AQUA score group and survival outcome within the IPI group. We classified patients into two groups based on IPI for further analysis: low IPI group (IPI 0–2) and high IPI group (IPI 3–5). In the entire cohort, 110 patients (33.1%) were classified in high IPI group (IPI 3–5). A relatively high proportion of patients with high BCL2 AQUA score were classified in the high IPI group (46.4% and 34.2%, *p* = 0.04894). There was a significant association between BCL2 AQUA score and survival outcomes within both low and high IPI groups (Fig. 4A,D). In addition, high BCL2 AQUA score was associated with poor prognosis within both GCB and non-GCB DLBCL, although there was only a marginal statistical significance regarding OS in non-GCB DLBCL (Fig. 4E,H).

**Figure 5:**
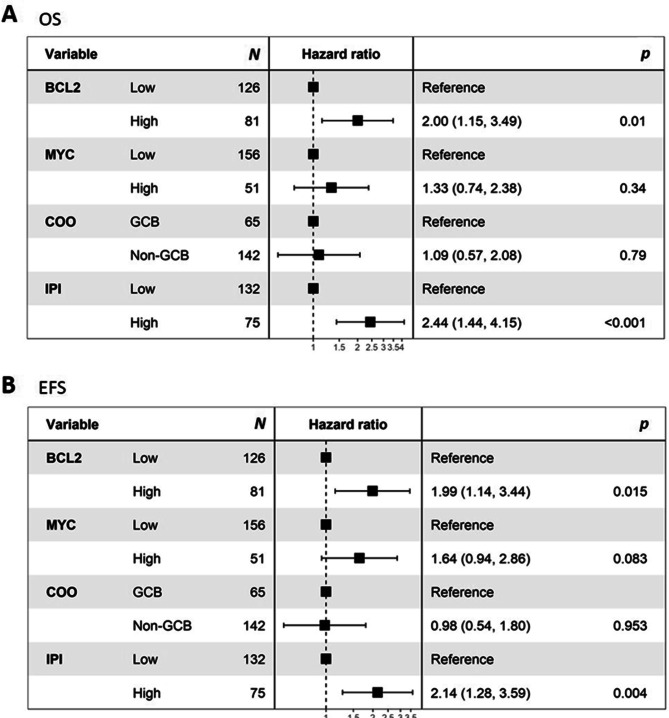
Forest plots for the adjusted hazard ratio for the BCL2 and MYC automated quantitative analysis (AQUA) scores and clinicopathologic factors. In multivariate analysis, high BCL2 AQUA score and high IPI group were associated with inferior overall survival (**A**) and event-free survival (**B**) independent of other clinicopathologic factors including MYC AQUA score and cell of origin (COO).

In the multivariate analysis, high BCL2 group was independently associated with poor OS (vs. low BCL2 group; HR 2.00; *p* = 0.01) and EFS (vs. low BCL2 group; HR 1.99; *p* = 0.015) in the training set (Fig. 5A,B). In addition, we further evaluated the prognostic value of BCL2 AQUA score as a continuous variable in the multivariate analysis, and it remained as an independent prognostic factor regarding both OS (HR: 1.016, *p* = 0.041) and EFS (HR: 1.016, *p* = 0.026) (Supplementary Table S3).

### Intensity incorporated analysis of BCL2 expression in single chromogenic IHC better predicts prognosis of DLBCL than the conventional method

According to the above-mentioned results, we implemented a BCL2 IHC analysis method incorporating both intensity and proportion. Of the existing IHC analysis methods, we decided to use the H-scoring system, which adds the results of the multiplication of the percentage of cells with the staining intensity ordinal value.

Of the entire cohort, 191 cases were available for the additional BCL2 IHC analysis. After normalizing between 0 and 100, a H-score of 66.73 was determined as the optimal cutoff value and 69 patients (36.1%) were classified as being in the high BCL2 group. Patients in the high BCL2 group were significantly associated with poor OS and EFS (*p* = 0.0011; OS, *p* = 0.013; EFS) (Supplementary Figure S5A,B). Subsequently, we compared the performance for predicting prognosis between the tumor-specific AQUA scoring system, tumor-specific conventional method, H-scoring system, and the conventional method. In ROC analysis, the H-scoring system generally showed similar prediction performance to the tumor-specific AQUA scoring system (AUROC = 0.63 for OS and 0.60 for EFS) (Supplementary Figure S5C,D).

## Discussion

BCL2 overexpression has been thought to provide a survival advantage for malignant B-cells, however, the prognostic impact of BCL2 expression in DLBCL is still controversial^21,25^. The discrepancies between different studies may originate from the semiquantitative and subjective interpretation of BCL2 expression by IHC. In the studies reported so far, various cutoff values (1–70%) have been used for the interpretation of BCL2 expression^32^. In addition, these cutoff values have been evaluated only in terms of the proportion of the positive staining cells and not for the various staining intensities often observed in clinical practice. A recent Japanese study proposed a new scoring system for the evaluation of BCL2 expression which included both the staining intensity and proportion^25^. Nevertheless, this scoring system was based on discrete cut-off values of the staining intensity and proportion. To the best of our knowledge, our present study is the first study to evaluate the prognostic value of a scoring system which objectively and quantitatively interprets both the staining intensity and proportion into a continuous variable. By using IF instead of chromogen for antigen detection, we generated a more linear output with a wider dynamic range^33^. In addition, multiplex IF staining enabled tumor-specific analysis which allowed for BCL2 expression in reactive T-cells to be easily excluded from the analysis. As a result, we confirmed the importance of incorporating the intensity data of BCL2 expression in risk stratification and also identified the high BCL2 AQUA score as an independent poor prognostic factor.

After clearly confirming that BCL2 AQUA score, which was based on both the staining intensity and proportion, is significantly associated with the survival outcome, we subsequently investigated a practical method of incorporating the staining intensity and proportion in the interpretation of BCL2 expression using IHC. Setting up a scoring strategy by the IHC system is important because IHC is a practically well-established and relatively low-cost method for detecting target protein expression^34^. We adopted the H-scoring system for BCL2 analysis in single chromogenic IHC and confirmed that patients with high BCL2 H-score were associated with poor prognosis. In addition, H-score could predict prognosis more robustly than conventional methods using threshold percentages. Moreover, the H-scoring method showed similar performance to the tumor-specific AQUA scoring system. However, careful observation was required not to include reactive T-cells when selecting the target regions for image analysis. This suggests that interobserver discrepancy may exist in H-scoring when using image analysis. Therefore, additional training might be required to achieve maximum performance while using the H-scoring system in single IHC.

Over the past decade, the COO-based subtype-specific approach has led most of the DLBCL studies in terms of prognosis and treatment. However, we believe that the current paradigm of treatment in DLBCL could be modified to be based on BCL2 status for the following reasons: (1) Recent studies suggested that BCL2/MYC expression can contribute to the inferior prognosis of ABC subtype DLBCL and showed that BCL2 genetic alterations could confer further risk stratification for both ABC and GCB subtype DLBCL^8^; (2) The selective and potent BCL-2 inhibitor, venetoclax, recently received the approval of the Food and Drug Administration in chronic lymphocytic leukemia^35^ and showed a remarkable overall response rate of 44% in the first-in-human study in patients with relapsed or refractory non-Hodgkin lymphoma^36^; (3) In vitro and in vivo studies using cell lines, primary patient samples, and patient-derived xenograft (PDX) mouse models demonstrated that high BCL2 levels were associated with extreme sensitivity to venetoclax and a lack of BCL2 was associated with resistance to venetoclax^37^. For these reasons, we believe that the clinical implications of BCL2 are growing and that the optimal evaluation of BCL2 expression is becoming more crucial in DLBCL.

We demonstrated that patients in the high BCL2 group were significantly associated with inferior outcome within both low and high IPI groups. The IPI is still an important factor in predicting the prognosis of DLBCL and is used in all clinical and research fields. In our study, the high IPI group also showed the highest hazard ratio in the multivariate analysis (HR 2.44, *p* < 0.001; OS, HR 2.14, *p* = 0.004; EFS). However, IPI has been considered to have limitations in predicting patients who will have a particularly aggressive outcome since the R-CHOP era^38^. Although many biological prognostic markers have been described, few have been utilized in clinical practice^39^ Finding new biological markers is essential for the addition of a novel agent in the current R-CHOP backbone and for creating an individualized care plan for patients with DLBCL. Taken together, a quantitative and reproducible analysis method such as the AQUA scoring system may be useful in the clinical application of biological prognostic markers.

In conclusion, BCL2 expression evaluated by AQUA scoring system incorporating both intensity and proportion of BCL2 was an independent prognostic factor in patients with DLBCL treated with R-CHOP. AQUA scoring system was demonstrated to be feasible and reproducible with a better performance than conventional method. Given the increasing clinical significance of BCL2 and considering therapeutic advances targeting BCL2 in hematologic malignancies, the specific definition of BCL2 positivity in DLBCL gives great promise to the study of pathophysiology of DLBCL and can be used to establish new therapeutic strategies.

## Supplementary information

Supplementary file1 (PDF 1797 kb)

## Data Availability

Due to strict data protection rules we cannot make the materials and data freely available. However, anonymous, aggregated datasets that can replicate the results might be considered disclosed upon request.
