# Supplementary material for: Quantitative analysis of tumor-specific BCL2 expression in DLBCL: refinement of prognostic relevance of BCL2
Source: Sci Rep. 2020 Jun 30;10:10680. doi: 10.1038/s41598-020-67738-4 (PMC7326926; doi:10.1038/s41598-020-67738-4)
Supplement: Supplementary file 1 — Supplementary file1 (PDF 1797 kb) [file 41598_2020_67738_MOESM1_ESM.pdf]

Quantitative analysis of tumor-specific BCL2 expression in DLBCL: refinement of prognostic relevance of BCL2

Jin Roh[1*], Hyungwoo Cho[2*], Dok Hyun Yoon[2], Jung Yong Hong[2], A-Neum Lee[3], Hyeon Seok Eom[4], Hyewon Lee[4], Weon Seo Park[4], Jae Ho Han[1], Seong Hyun Jeong[5], Joon Seong Park[5], Hyo-Kyung Pak[3], So-Woon Kim[6], Sang-Yeob Kim[7], Cheolwon Suh[2], Jooryung Huh[6], and Chan-Sik Park[3,6]

[1]Department of Pathology, Ajou University School of Medicine, Suwon, Korea; [2]Department of Oncology, University of Ulsan College of Medicine, Asan Medical Center, Seoul, Korea; [3]Asan Institute for Life Science, University of Ulsan College of Medicine, Asan Medical Center, Seoul, Korea; [4]Center for Hematologic Malignancy, National Cancer Center, Goyang, Korea; [5]Department of Hematology-Oncology, Ajou University School of Medicine, Suwon, Korea; [6]Department of Pathology, University of Ulsan College of Medicine, Asan Medical Center, Seoul, Korea; and [7]Department of Convergence Medicine, University of Ulsan College of Medicine, Seoul, Korea

Supplementary tables

Supplementary Table S1. Baseline characteristics of patients with diffuse large B-cell lymphoma in the validation set.

Characteristics	*N* (total = 111)	%	
Age > 60y	52	46.8	
Sex, male	63	56.8	
LDH > normal	36	36	*N* = 100
B symptoms	5	14.7	*N* = 34
Stage 3-4	49	46.7	*N* = 105
IPI 3-5	29	35.4	*N* = 82

LDH: Lactate dehydrogenase; IPI: International Prognostic Index

Supplementary Table S2. Univariate analysis of proportion of BCL2 positive cells, BCL2 intensity, and BCL2 AQUA score as a continuous variable for overall survival and event-free survival.

	Overall survival			Event-free survival		
Variable	HR	95% CI	*p* value	HR	95% CI	*p* value
Proportion of BCL2 positive cells	1.012	1.005–1.020	0.002	1.012	1.005–1.019	0.001
BCL2 intensity	1.000	1.000–1.000	0.015	1.000	1.000–1.000	0.126
BCL2 AQUA score	1.022	1.01–1.034	<0.001	1.020	1.009–1.033	<0.001

Supplementary Table S3. Multivariate analysis of AQUA scores of BCL2 and MYC as a continuous variable for overall survival and event-free survival.

	Overall survival			Event-free survival		
Variable	HR	95% CI	*p* value	HR	95% CI	*p* value
BCL2	1.016	1.001–1.031	0.041	1.016	1.002–1.029	0.026
MYC	1.000	0.986–1.020	0.772	1.003	0.988–1.019	0.686
IPI	1.022	1.252–1.795	<0.001	1.374	1.167–1.619	<0.001
non-GCB (vs. GCB)	1.025	0.551–1.907	0.937	0.920	0.533–1.590	0.766

Supplementary Figures

Supplementary Figure S1. Multiplex immunofluorescence analysis of BCL2 in diffuse large B-cell lymphoma (DLBCL). (A) Composite image of CD20-Opal 650 (green), BCL2-Opal 520 (yellow), CD3-Opal 570 (orange), MYC-Opal 690, and DAPI (blue). (B) Segmentation of subcellular compartments. (C) CD20-Opal 650 image is used to define the tumor. CD20-Opal650 stain shows membranous pattern. D) BCL2-Opal 520 stain shows cytoplasmic pattern.

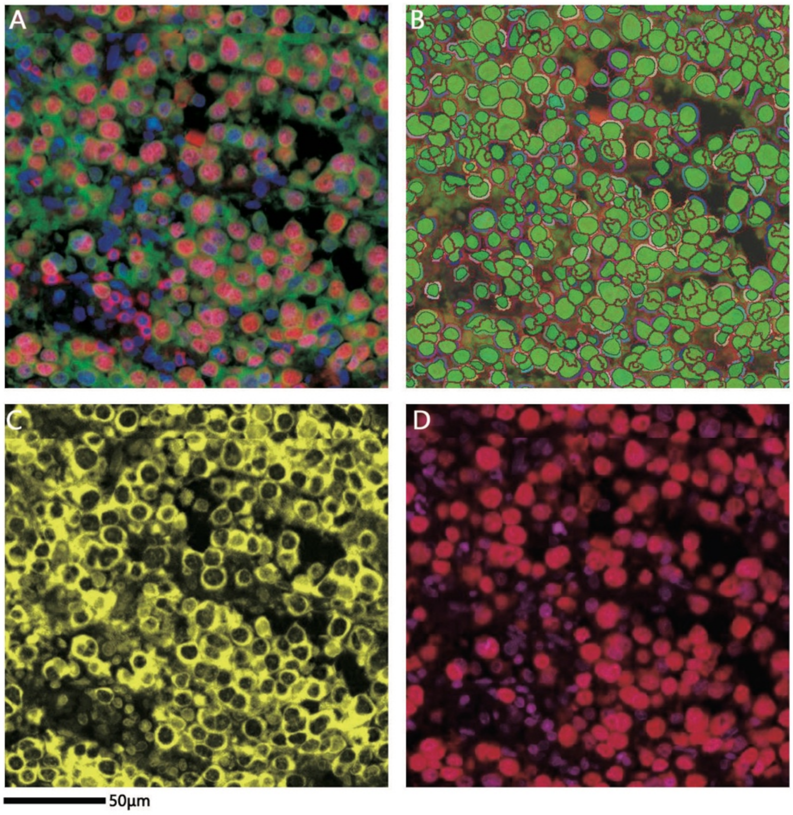

Supplementary Figure S2. Various BCL2 intensities in the single chromogenic BCL2 IHC.

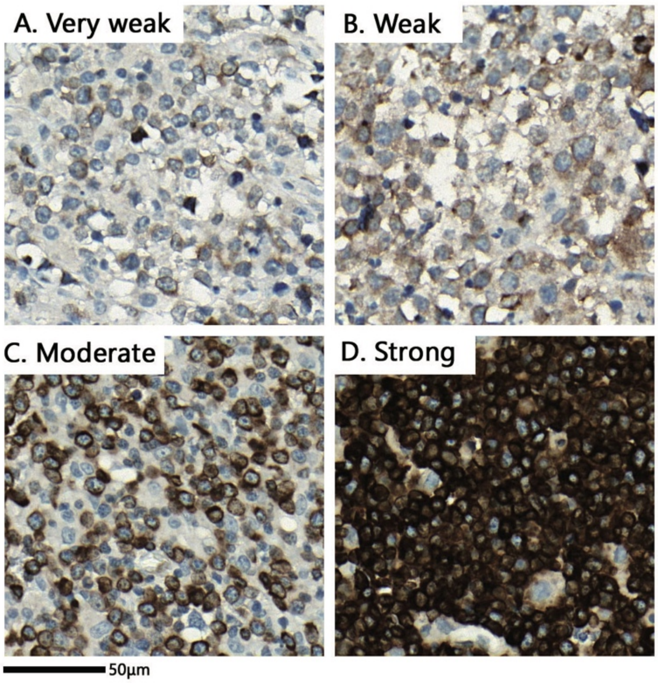

IHC: Immunohistochemistry

Supplementary Figure S3. Distribution of BCL2 AQUA scores according to the clinical conditions. The AQUA scores were compared in various clinical conditions representing the prognosis of patients with DLBCL. Patients with poor clinical conditions such as no complete response for initial R-CHOP treatment (A, p = 0.01053), death in the entire observation time (B, p = 0.0008803), death within 5 years of diagnosis (C, p = 0.0008853), clinical events within 2 years of diagnosis (D, p = 0.000316) showed significantly low AQUA score in the t-test.

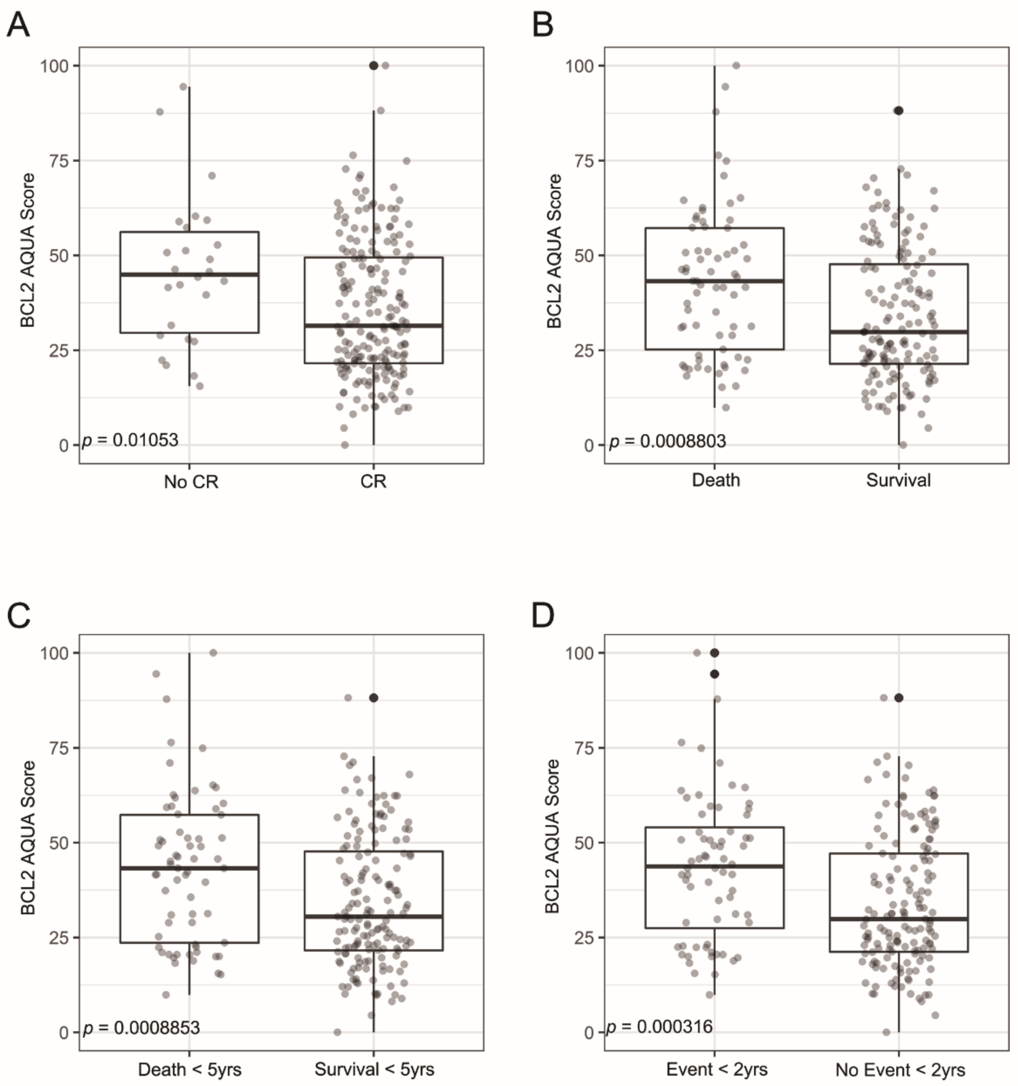

AQUA: Automated Quantitative Analysis

CR: Complete response

OS: Overall survival

EFS: Event-free survival

Supplementary Figure S4. Survival analysis with an increase in the BCL2 AQUA score. (A-B) OS (A) and EFS (B) according to the BCL2 AQUA score in the training set. Survival curves showed the worse outcomes with increasing the BCL2 AQUA score. (C-D) Forest plots for the hazard ratio according to the BCL2 AQUA score in the training set. For OS and EFS, forest plots also showed a higher hazard rate as the BCL2 AQUA score increases.

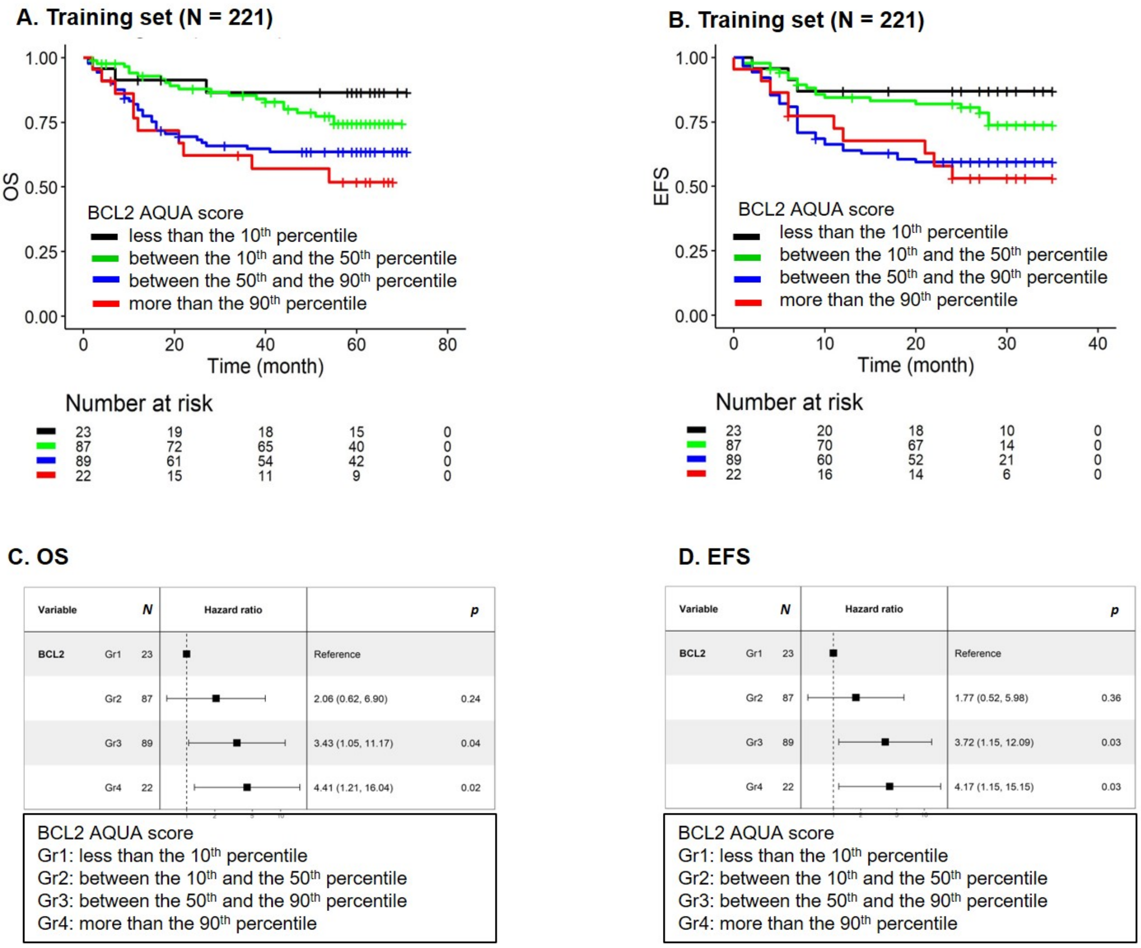

AQUA: Automated Quantitative Analysis

OS: Overall survival

EFS: Event-free survival

Supplementary Figure S5. Survival analysis according to the H-score of BCL2 IHC and comparison of prediction performance between methods for BCL2 analysis. (A-B) Survival curves according to the H-score of BCL2 IHC showed that the high BCL2 H-score was significantly associated with inferior OS (A) and EFS (B). ROC curves for OS (C) and EFS (D) according to methods for BCL2 analysis revealed that the H-scoring system for BCL2 IHC had similar prediction performance to the BCL2 AQUA scoring system.

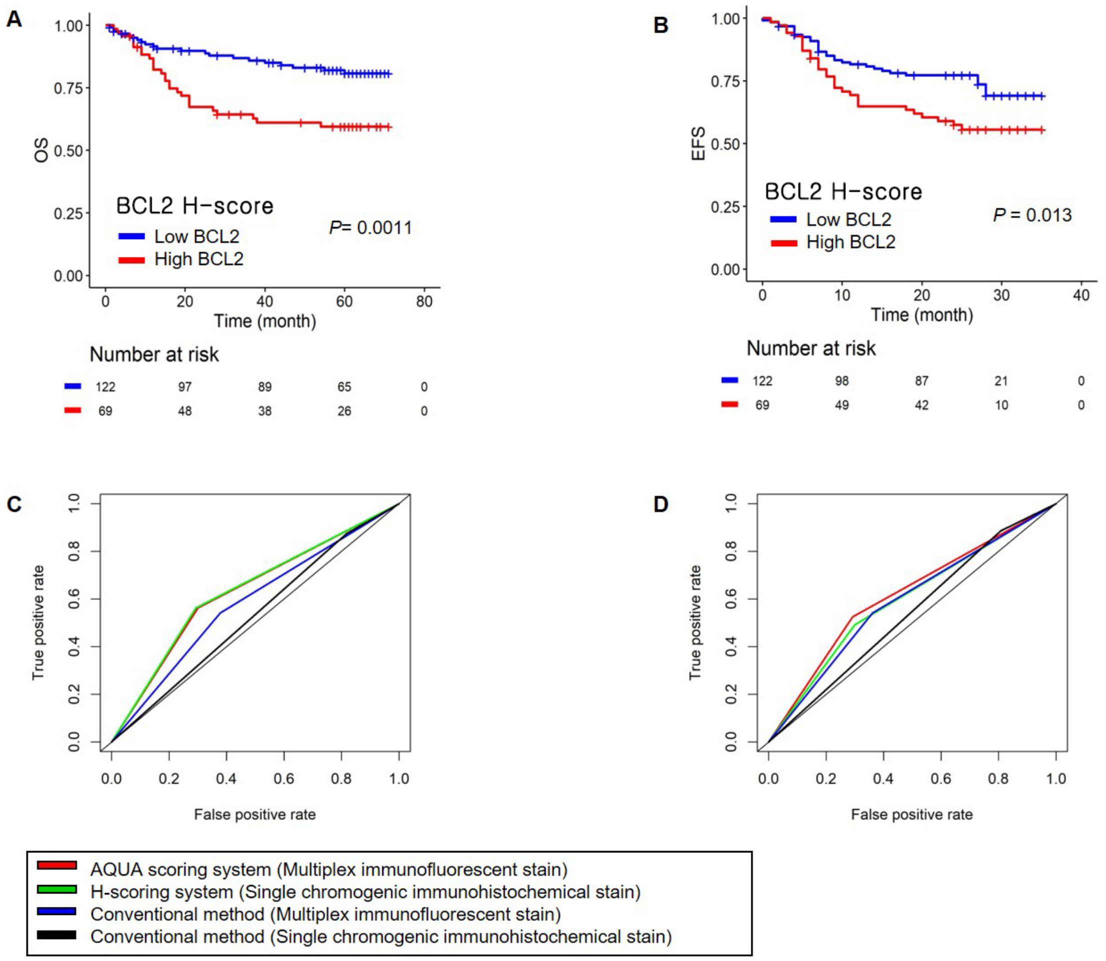

ROC: Receiver operating characteristic

AQUA: Automated Quantitative Analysis

IHC: Immunohistochemistry

OS: Overall survival

EFS: Event-free survival
